# Analysis of the prevalence of allergens listed in European baseline series in products composition used in atopic dermatitis available in Polish online drugstores

**DOI:** 10.1186/2045-7022-5-S1-O12

**Published:** 2015-03-11

**Authors:** Katarzyna Osinka, Agnieszka Mysliwiec, Wojciech Feleszko, Agnieszka Krauze

**Affiliations:** 1 Medical University of Warsaw, Pediatric Pulmonology and Allergology Clinic, Warsaw, Poland

## Background

Atopic dermatitis (AD) is a chronic, recurrent inflammatory skin disease. Due to deficient skin barrier function, patients with AD are exposed to higher risk of contact sensitization and allergic contact dermatitis. Basic therapy of AD should consist of optimal skin care pointed at the skin barrier defect with regular use of emollients. Unfortunately, products from this group can potentially comprise compounds listed in European Baseline Series (EBS) used in the patch test allergens. The aim of this study was to determine the prevalence of allergens listed in European Baseline Series included in preparations available in polish online drugstores.

## Method

About 40 online drugstores were searched by two independent researchers. We included preparations described by the manufacturers as "emollients" or "products able for treatment of atopic dermatitis" and subsequently assessed for their presence of chemical compounds included to list of the EBS(28 most common allergens). The analysis also included the cost of each product (all collected on the same day, 28 Jan 2014).

## Results

196 preparations met our inclusion criteria. 17 of them (8,7%) weren't examined because of the lack of information about ingredients based on INCI (International Nomenclature of Cosmetics Ingredients). The analysis of the data revealed that 60 (58,3%) emollient products contained Parabens, 25 (24,3%)- Wool alcohols, 17 (16,5%)- Fragrance mix I, 7(6,8%)- 5-chloro-2-methyl-4-isothiazolin-3-one (Cl+Me-isothiazolinone), 10(9,7%)- Formaldehyde, 9 (8,7%)- Fragrance mix II. 49 out of them (47,6%) contained fragrances not included in EBS. Among 179 preparations, only 71(39,7%) met the criteria of ingredients deprived of allergens listed in EBS. Our study showed that 36 (50,7%) out of them were more expensive than 50z³ per 200 ml, whereas 35 (49,3%) of them were cheaper.

## Conclusion

Current analysis of composition of emollient preparations demonstrated that majority of them contained allergens listed in EBS. There was no correlation between high price of preparation and allergen deprived composition. In view of increasing incidence of atopic dermatitis in children, a wide range of emollient preparations is observed. This is a matter of public concern not to allow products used in AD to do harm to children. This study underscores that additional chemical compounds in emollient preparations are avoidable risk factor for the development of contact dermatitis in children.

